# Chromosome level genome assembly of endangered medicinal plant *Anisodus tanguticus*

**DOI:** 10.1038/s41597-024-03007-7

**Published:** 2024-02-02

**Authors:** Yongli Song, Jian-Ping Huang, Yong-Jiang Wang, Sheng-Xiong Huang

**Affiliations:** 1 State Key Laboratory of Southwestern Chinese Medicine Resources, School of Pharmacy, Chengdu University of Traditional Chinese Medicine, Chengdu, 611137 China; 2 State Key Laboratory of Phytochemistry and Plant Resources in West China, Kunming Institute of Botany, Chinese Academy of Sciences, Kunming, 650201 China

**Keywords:** Genome, Plant evolution

## Abstract

*Anisodus tanguticus* is a medicinal herb that belongs to the *Anisodus* genus of the Solanaceae family. This endangered herb is mainly distributed in Qinghai–Tibet Plateau. In this study, we combined the Illumina short-read, Nanopore long-read and high-throughput chromosome conformation capture (Hi-C) sequencing technologies to *de novo* assemble the *A. tanguticus* genome. A high-quality chromosomal-level genome assembly was obtained with a genome size of 1.26 Gb and a contig N50 of 25.07 Mb. Of the draft genome sequences, 97.47% were anchored to 24 pseudochromosomes with a scaffold N50 of 51.28 Mb. In addition, 842.14 Mb of transposable elements occupying 66.70% of the genome assembly were identified and 44,252 protein-coding genes were predicted. The genome assembly of *A. tanguticus* will provide genetic repertoire to understand the adaptation strategy of *Anisodus* species in the plateau, which will further promote the conservation of endangered *A. tanguticus* resources.

## Background & Summary

The perennial medicinal herb *Anisodus tanguticus* is a member of *Anisodus* genus that is distributed in Qinghai–Tibet Plateau. *A. tanguticus* was named as “Tang Chun Na Bao” in the traditional Tibetan medicine^1^. Its roots were used by the local Tibetan healers to treat septic shock, ulcers, colitis, spasms and reduce pain^1,2^. The main active components of *A. tanguticus* roots are tropane alkaloids, such as hyoscyamine, anisodamine, and scopolamine^3^. These tropane alkaloids are the competitive, reversible antagonists of muscarinic acetylcholine receptors, and are clinically used for the treatment of motion sickness, spasticity, obstetrical analgesia, septic shock, organophosphate poisoning, Parkinson’s symptoms, etc^2,4^. Besides, atropine (racemic hyoscyamine) was listed as the most efficacious, safe, and cost-effective medicines for priority conditions in the World Health Organization model list of essential medicines (https://www.who.int/publications/i/item/WHO-MHP-HPS-EML-2021.02). In addition to the well-known tropane alkaloids, numerous terpenoids, indolizidine- and pyrrolidine-type alkaloids and cinnamoylphenethylamides with pharmacological activity have been isolated from *A. tanguticus*^5–8^. Due to the important medicinal value, *A. tanguticus* has been massively exploited and collected, resulting in the depletion of its wild resources.

In the *Anisodus* genus, there are four species and three varieties, such as *A. tanguticus*, *A. luridus*, *A. acutangulus*, and *A. mairei*^9^. These four species are mainly distributed in the plateau (mainly the Qinghai–Tibet Plateau) at altitudes ranging from 2,680 to 4,200 m, and *A. tanguticus* was observed to survive at a higher altitude environment than *A. acutangulus*^9^. Although the genome of *A. acutangulus* has been assembled to explore the evolution of tropane alkaloid biosynthesis^10^, few is known about the adaptation strategy of *Anisodus* species to overcome the adverse environment, such as the complex land conditions or the diverse climate. Recently, the chloroplast genome of *A. tanguticus* was sequenced to study the adaptation strategy of *A. tanguticus* in the Qinghai–Tibet Plateau^11,12^. The chloroplast genetic information accounts for only a small part of the whole genetic information of *A. tanguticus*, and most genetic information is deposited within the chromosomal DNA. Thus, a high-quality chromosomal-level genome is necessary to provide genetic information to understand the evolutionary process of the *Anisodus* genus and the adaptation strategy of *Anisodus* species in the plateau, which will also promote the conservation of endangered *A. tanguticus* resources.

**Fig. 1:**
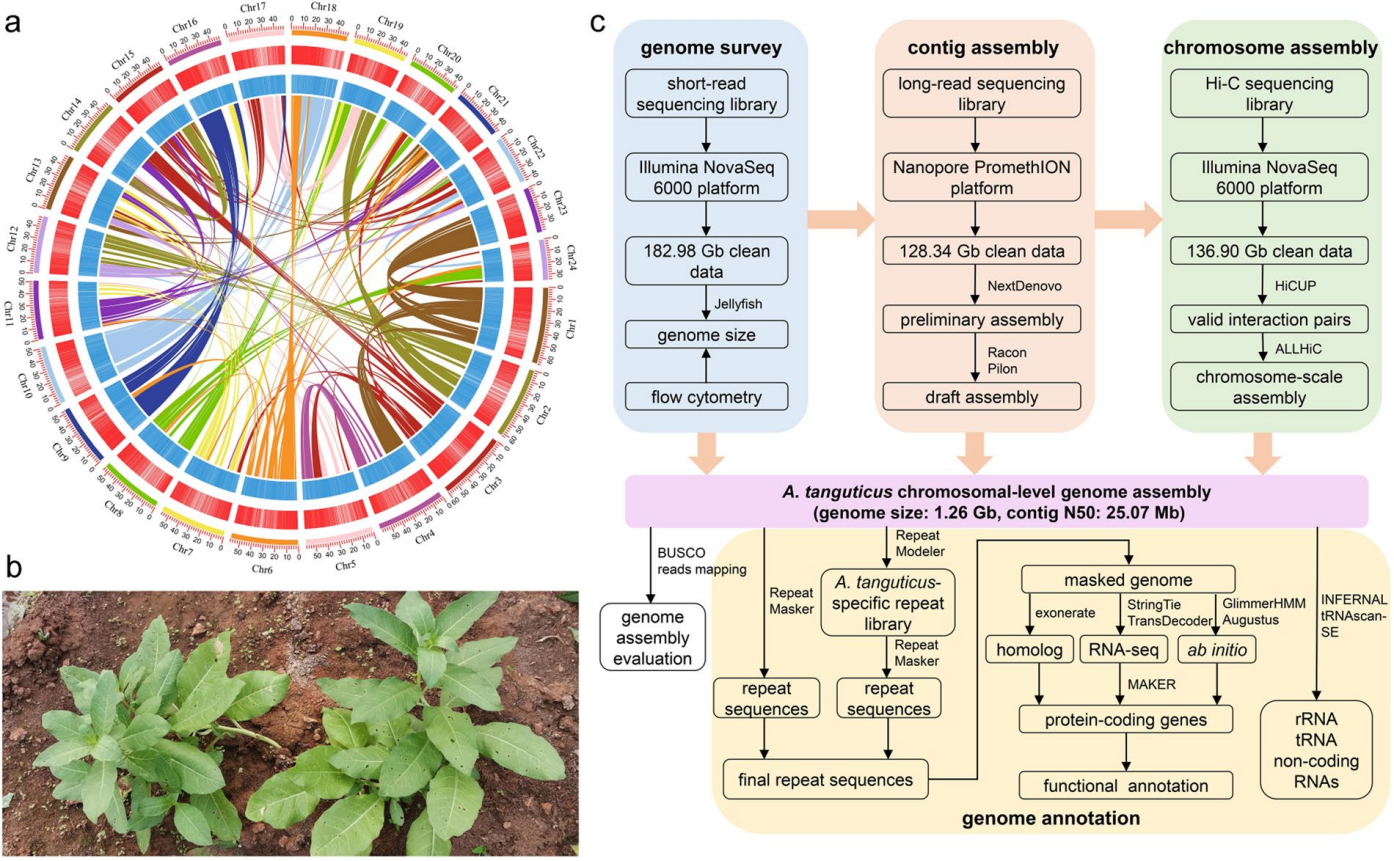
The genome assembly and annotation of *A. tanguticus*. (**a**) Circular map of *A. tanguticus*. The 24 outer lines represent 24 pseudochromosomes (Chr1−24). The blue and red bands represent the density of transposable elements and protein-coding genes, respectively. The inner lines represent syntenic blocks in the *A. tanguticus* assembly. (**b**) Photograph of *A. tanguticus*. (**c**) The process pipeline of *A. tanguticus* genome assembly and annotation.

In this paper, we generated a high-quality chromosomal-level genome assembly of *A. tanguticus* based on the Illumina short-read sequencing (182.98 Gb), Nanopore long-read sequencing (128.34 Gb) and Hi-C sequencing (136.90 Gb). The assembled genome, composed of 276 contigs, had a genome size of 1.26 Gb with a contig N50 of 25.07 Mb (Table 1). These contigs were anchored to 24 pseudochromosomes, with an anchoring rate of 97.47% and a scaffold N50 of 51.28 Mb (Table 1, Fig. 1). Of this genome assembly, 66.70% (842.14 Mb) were transposable elements with a major component of long terminal repeats (LTRs), which accounted for 44.51% (Tables 1, 2). Meanwhile, 44,252 protein-coding genes composed the final gene repertoire of *A. tanguticus* (Table 1). This high-quality genome will provide a genetic basis for understanding the adaptive evolution of *A. tanguticus* in the plateau.

**Table 1:** Genome assembly and annotation statistics for *A. tanguticus*.

Genome assembly statistics	
Genome size (bp)	1,262,533,339
Number of chromosomes	24
Genome size in chromosomes (bp)	1,230,579,671
Genome in chromosomes (%)	97.47
Number of contigs	276
Contig N50 (bp)	25,065,403
Number of scaffolds	131
Scaffold N50 (bp)	51,279,509
Number of protein-coding genes	44,252
Average gene/CDS length (bp)	6,868/1,155
Total size of Repeat sequences (bp)	842,143,897
Repeat sequences in genome (%)	66.70

**Table 2:** Summary of repeat contents in *A. tanguticus*.

Type	TE proteins		*De novo* + Repbase		Combined TEs	
	Length (bp)	Ratio (%)	Length (bp)	Ratio (%)	Length (bp)	Ratio (%)
DNA	2,777,268	0.22	112,613,118	8.92	112,839,725	8.94
LINE	17,913,877	1.42	43,874,441	3.48	48,309,924	3.83
SINE	0	0.00	2,595,535	0.21	2,595,535	0.21
LTR	257,802,593	20.42	550,850,320	43.63	561,967,312	44.51
LTR-*Gypsy*	50,267,778	3.98	199,921,575	15.83	204,019,329	16.16
LTR-*Copia*	198,781,093	15.74	327,343,555	25.93	331,645,793	26.27
Satellite	0	0.00	915,584	0.07	915,584	0.07
Other	591	0.00	3,315	0.00	3,906	0.00
Unknown	17,115	0.00	123,903,677	9.81	123,920,792	9.82
Total	278,491,062	22.06	815,145,898	64.56	842,143,897	66.70

## Methods

### Sample collection and genomic DNA extraction

The seeds of *A. tanguticus* were collected from Qilian, Qinghai Province, China, and stored in the Germplasm Bank of Wild Species in Southwest China. *A. tanguticus* plants were cultivated in the Kunming Institute of Botany of the Chinese Academy of Sciences, Yunnan Province, China. Young leaves from an individual *A. tanguticus* plant were collected and then used for genomic DNA (gDNA) extraction following the modified cetyltrimethylammonium bromide (CTAB) protocol^13^. The purity and quality of extracted gDNA were examined by NanoPhotometer spectrophotometer (Implen, USA) and agarose gel electrophoresis. Three different tissue samples, including leaf, stem, and root, were collected from an individual cultivated *A. tanguticus* plant, and used for RNA extraction.

### Illumina sequencing and genome survey analysis

High-quality gDNA was randomly fragmented by ultrasonic oscillation (Covaris, USA) and used for Illumina short-read sequencing. According to the protocol of TruSeq DNA Sample Preparation Guide (Illumina, USA), the sequencing libraries were constructed with 350 bp insert size. Then, these libraries were sequenced on the Illumina NovaSeq 6000 platform (Illumina, USA) with a mode of paired-end 150 bp at Benagen Technology Co., Ltd. (Wuhan, China). After removing low-quality reads, the resulting 182.98 Gb clean data were used for the survey analysis of *A. tanguticus* genome and the polish of preliminary assembly.

**Fig. 2:**
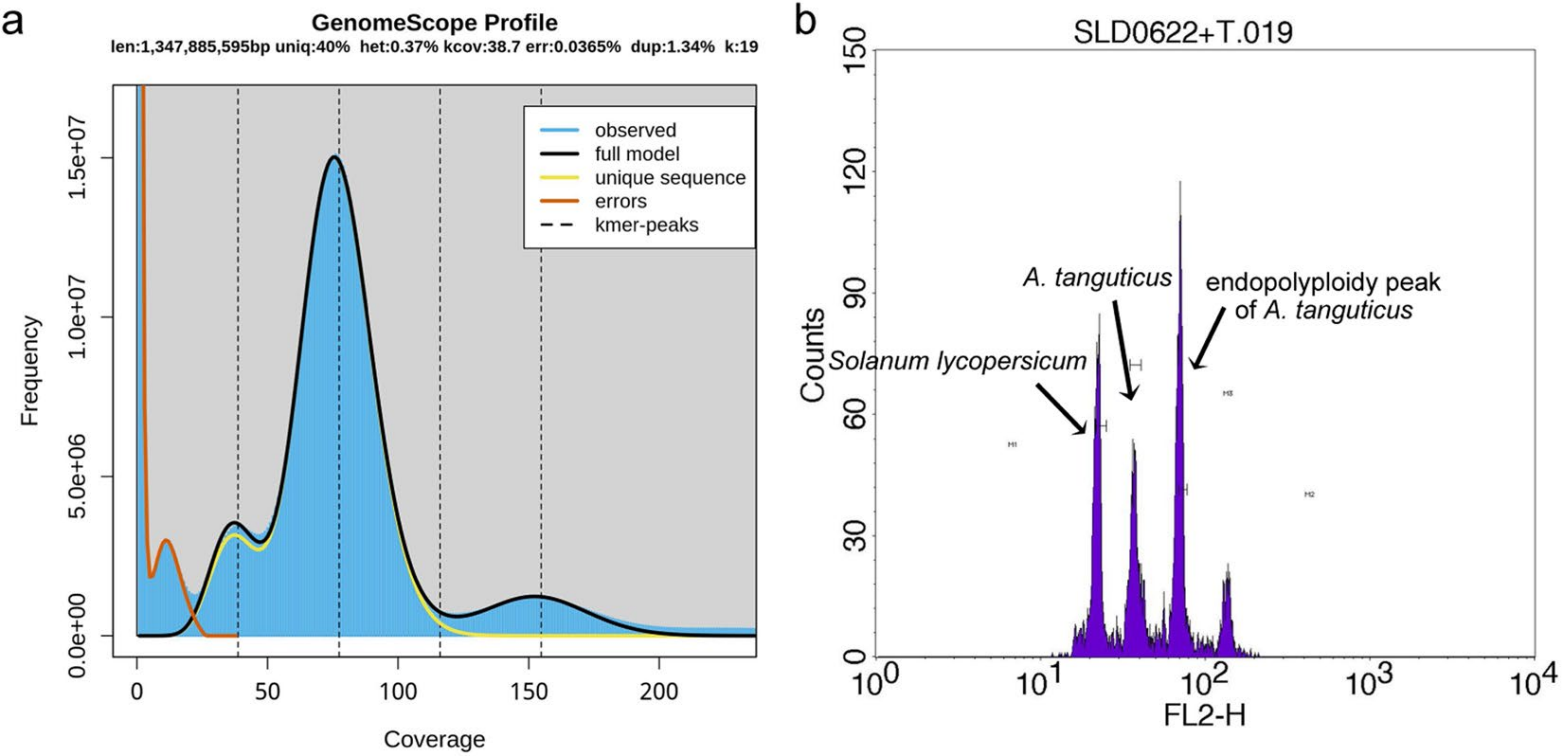
The evaluation of *A. tanguticus* genome size. (**a**) Genome scope profiles of 19-mer analysis. The X-axis represented the k-mer depth and the Y-axis represented the frequency of the k-mer for a given depth. (**b**) The flow cytometry of *A. tanguticus*. Endopolyploidy was observed in the genome of *A. tanguticus*.

The frequencies of 19-kmer were generated by Jellyfish (version 2.2.10) based on the clean data and used for the genome evaluation by GenomeScope (version 2.0) (Fig. 2a)^14,15^. As a result, the genome size of *A. tanguticus* was estimated as 1.35 Gb, which was consistent with the genome size (~1.5 Gb) measured by flow cytometry (Fig. 2b). Meanwhile, the heterozygous ratio and the repeat content were estimated as 0.37% and 60.0%, respectively.

### Nanopore sequencing and draft genome assembly

For nanopore long-read sequencing, its libraries were constructed under the protocol of SQK-LSK110 Ligation Sequencing Kit (Nanopore, UK). The prepared libraries were loaded on flow cells (R9.4) and sequenced on the Nanopore PromethION platform (Nanopore, UK). After removing low-quality reads, a total of 128.34 Gb of clean data, composed of 8.22 million reads, were obtained. The N50 read length was 32.63 kb and the longest nanopore read length was 394.22 kb.

The preliminary assembly was generated by NextDenovo (https://github.com/Nextomics/NextDenovo) with 128.34 Gb clean nanopore data. Subsequently, Racon (version: 1.4.11)^16^ was used to polish the preliminary assembly with nanopore long-reads through two iterations. Pilon (version: 1.23)^17^ was used to polish the preliminary assembly with Illumina short-reads through two iterations. As a result, the draft genome of *A. tanguticus* was assembled with a total length of 1.26 Gb, composed of 276 contigs and the contig N50 was 25.07 Mb (Table 1).

### Hi-C sequencing and chromosome-scale assembly

For genome scaffolding, the fresh leaves were used to construct the Hi-C libraries according to the standard library preparation protocol^18^. The prepared libraries were sequenced on the Illumina NovaSeq 6000 platform (Illumina, USA) with a 150-bp paired-end strategy. After the filtration of raw data, 136.90 Gb of clean data were generated.

**Fig. 3:**
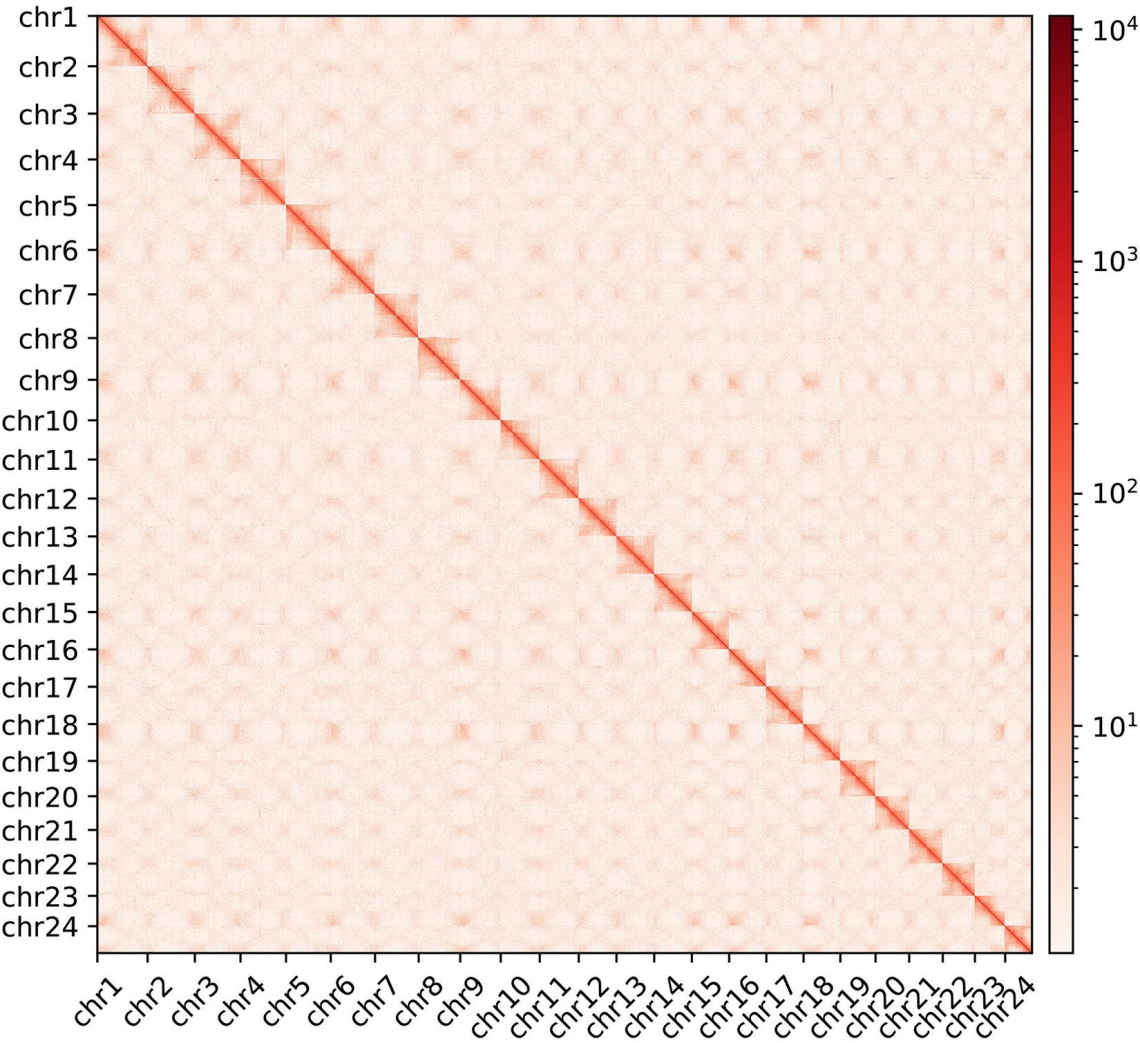
The Hi-C interaction heatmap of *A. tanguticus* genome. The dark red indicates high chromatin interactions, which were quantified based on the count of supporting Hi-C reads.

The valid interaction pairs were identified by HiCUP (version: 0.8.0) and used to construct chromosome-scale assemblies by ALLHiC (version: 0.9.8)^19,20^. Finally, 97.47% of the draft genome sequences (1.23 Gb) were anchored to 24 pseudochromosomes of *A. tanguticus* and the final chromosome-scale assembly was composed of 131 scaffolds with a scaffold N50 of 51.28 Mb (Table 1, Fig. 3).

### Genome annotation

Repeat sequences were identified by combining homology-based predictions and *ab initio* predictions. Firstly, RepeatMasker (version: 4.0.9) was used for homology-based prediction of the repeat sequences [i.e. “TE (transposable element) proteins” column in Table 2] in the genome assembly based on the Repbase database^21,22^. Secondly, RepeatModeler (version: 1.0.11) was used for *ab initio* prediction of the repetitive sequences to construct a *A. tanguticus*-specific repeat library^23^. This library was also used to annotate the repeat sequences (i.e. “*De novo* + Repbase” column in Table 2) of genome assembly by RepeatMasker (version: 4.0.9)^21^. These two repeat sequences were combined to obtain the final repeat sequences (i.e. “Combined TEs” column in Table 2), which accounted for 66.70% of the genome assembly.

Protein-coding genes were predicated by a combination of transcriptome-based prediction, *ab initio* predication and homologous predication. For transcriptome-based prediction, the RNA of three different tissues, including leaf, stem, and root, were used for the RNA sequencing. Stringtie (version: 2.1.4) and TransDecoder (version: 5.1.0, https://github.com/TransDecoder/TransDecoder) were used to predict the transcriptome-based genes^24^. GlimmerHMM (version: 3.0.4) and Augustus (version: 3.3.2) were used for the *ab initio* prediction^25,26^. Exonerate (version: 2.4.0) was used for homologous gene prediction with genes from *Solanum lycopersicum* (Sly), *Capsicum annuum* (Can), *Nicotiana attenuate* (Nat) and *Solanum tuberosum* (Stu)^27^. These predicated genes were integrated into 44,282 genes by MAKER (version: 2.31.10, Table 3)^28^. These protein-coding genes were annotated with protein sequence databases, including universal protein (Uniprot)^29^, protein families database (Pfam)^30^, gene ontology (GO)^31^, Kyoto encyclopedia of genes and genomes (KEGG)^32^, KEGG pathway database, interproscan database^32^, and nonredundant protein sequence (NR, https://www.ncbi.nlm.nih.gov/refseq/about/nonredundantproteins). 97.36% of protein-coding genes (43,112 genes) were annotated by at least one database (Table 4). In addition, 30 predicted genes with an intron less than 10 bp were designated as pseudogenes and eliminated in the gene repertoire of *A. tanguticus*, which led to a final gene count of 44,252.

**Table 3:** Statistical analysis of the gene structure of *A. tanguticus* genome.

Method	Gene set	Gene number	Average gene length (bp)	Average CDS length (bp)	Average exon length (bp)	Average intron length (bp)
*Ab initio*	GlimmmerHMM	95,347	12,708.31	796.87	180.30	3,483.33
*Ab initio*	AUGUSTUS	55,178	4,879.07	1,132.34	215.93	882.8
Homology-based	Sly	109,982	15,782.23	701.93	242.05	7,937.26
Homology-based	Can	108,692	12,305.26	687.06	238.25	6,167.49
Homology-based	Nat	107,513	26,712.07	771.54	253.39	12,685.38
Homology-based	Stu	113,516	14,452.74	661.54	246.91	8,212.57
RNAseq	TransDecoder	25,889	6,961.1	1,181.66	320.44	930.84
Integration	Maker	42,191	7,923.69	1,187.89	249.24	1,335.47
Final set	Anno-self	44,282	6,868.32	1,155.38	277.99	1,194.4

**Table 4:** Statistical analysis of the gene annotations of *A. tanguticus* genome.

Database	number	Ratio (%)
Annotation	43,112	97.36
Uniprot	42,013	94.88
Pfam	35,268	79.64
GO	34,928	78.88
KEGG	18,493	41.76
Pathway	9,806	22.14
Interproscan	41,763	94.31
NR	41,902	94.63
All	44,282	

The rRNA genes were predicated with rRNA database and the tRNA genes were predicated by tRNAscan-SE (version: 1.23)^33^. The non-coding RNAs were predicated by INFERNAL (version: 1.1.2) based on the Rfam database^34,35^. Finally, 2,758 tRNAs, 898 rRNAs, 1,821 snRNAs and 269 miRNAs were identified in *A. tanguticus*.

### Genome evolution

**Fig. 4:**
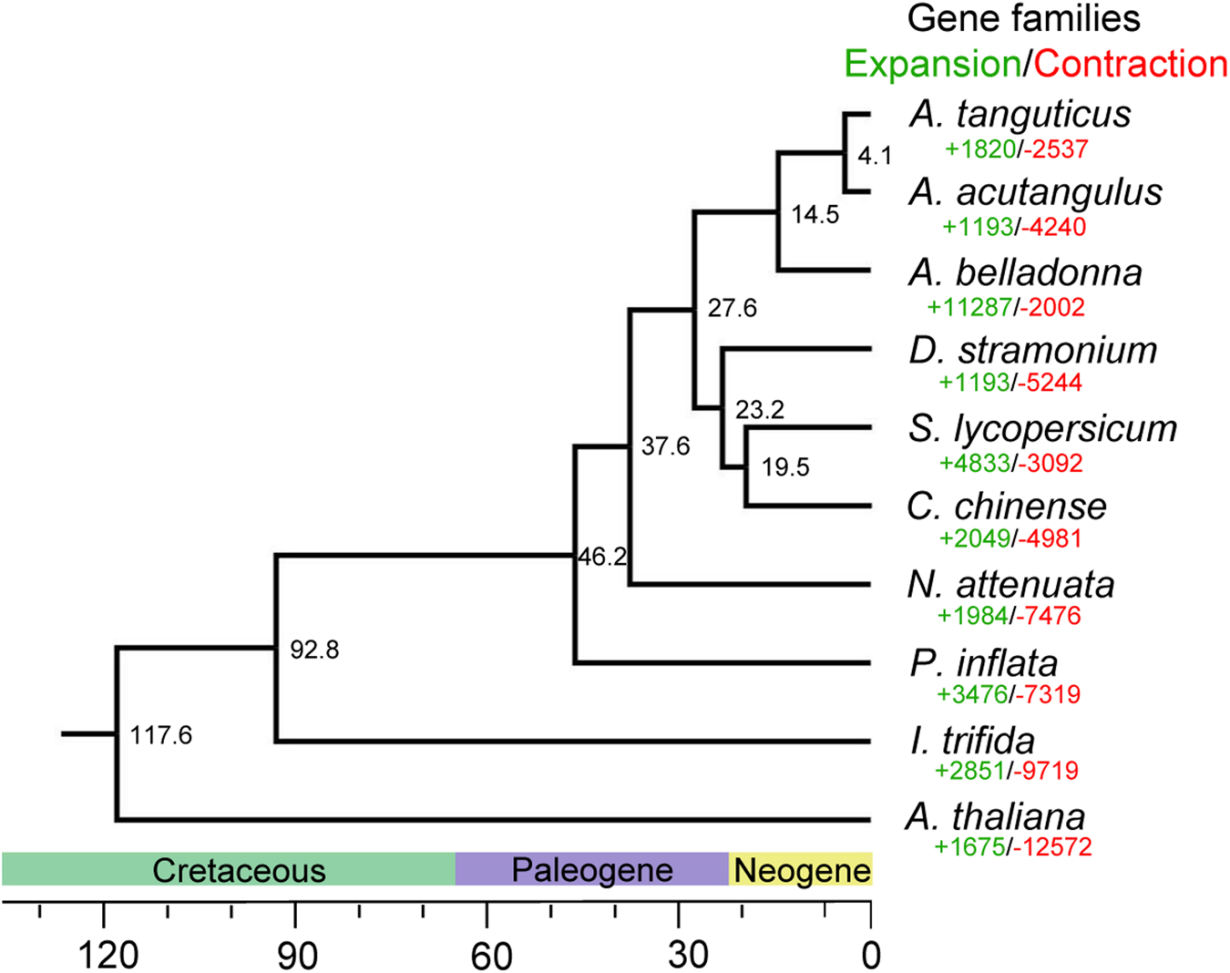
The inferred phylogenetic tree of *A. tanguticus* and nine other species. *A. tanguticus* and *A. acutangulus* clustered together.

175 single-copy orthologous families were clustered from *A. tanguticus*, *A. acutangulus*^10^, *Atropa belladonna*^36^, *Datura stramonium*^36^, *S. lycopersicum*^37^, *Capsicum chinense*^38^, *N. attenuate*^39^, *Petunia inflata*^40^, *Ipomoea trifida*^41^ and *Arabidopsis thaliana* by OrthoFinder (version: 2.5.2)^42^. These single-copy orthologous sequences were merged and aligned by MAFFT (version: 7.475)^43^. After the correction by Gblocks (version: 0.91b)^44^, the obtained sequences were used to construct the maximum likelihood tree by IQ-TREE (version: 2.0.3)^45^ with the best-fit model JTT + F + R3. The divergence time in the constructed phylogenetic tree was deduced by MCMCtree program (version: 4.9)^46^ with the divergence time of *A. tanguticus* and *A. thaliana* (111–124 Mya) from the TimeTree database (http://www.timetree.org). As a result, the divergence time between *A. tanguticus* and *A. acutangulus* was approximately 4.1 Mya (Fig. 4).

Based on the analysis of constructed phylogenetic tree and clustered gene families, 1820 and 2537 gene families were expanded and contracted in the *A. tanguticus* genome by CAFE analysis (version: 4.2.1)^47^, respectively (Fig. 4). Of these, 161 expanded gene families and 42 contracted gene families were statistically significant (Table 5). The significantly expanded 161 gene families were enriched in 38 GO terms, involved in “DNA metabolic process”, “DNA integration” and “mitochondrion” (Table 6), which were probably related to strong UV radiation and low temperature in the plateau.

**Table 5:** Summary of expanded and contracted gene families among *A. tanguticus* and nine other species.

Species	Number of expanded gene families	Number of contracted gene families	Number of significantly expanded gene families	Number of significantly contracted gene families
*A. tanguticus*	1,820	2,537	161	42
*A. acutangulus*	1,193	4,240	46	310
*A. belladonna*	11,287	2,002	351	7
*D. stramonium*	1,193	5,244	149	40
*S. lycopersicum*	4,833	3,092	189	69
*C. chinense*	2,049	4,981	238	50
*N. attenuata*	1,984	7,476	156	16
*P. inflata*	3,476	7,319	118	10
*I. trifida*	2,851	9,719	16	3
*A. thaliana*	1,675	12,572	4	1

**Table 6:** GO enrichment analysis of the significantly expanded gene families in *A. tanguticus*.

Class	GO_ID	GO_Name	P_value
Cellular component	GO:0005739	mitochondrion	7.38E-07
Cellular component	GO:0031966	mitochondrial membrane	0.010164
Cellular component	GO:0005740	mitochondrial envelope	0.012086
Cellular component	GO:0031967	organelle envelope	0.012975
Cellular component	GO:0031975	envelope	0.012975
Cellular component	GO:0098796	membrane protein complex	0.01886
Cellular component	GO:0009536	plastid	0.030632
Biological process	GO:0006259	DNA metabolic process	9.13E-09
Biological process	GO:0015074	DNA integration	1.09E-08
Biological process	GO:0034641	cellular nitrogen compound metabolic process	3.07E-05
Biological process	GO:0006139	nucleobase-containing compound metabolic process	8.81E-05
Biological process	GO:0006725	cellular aromatic compound metabolic process	1.95E-04
Biological process	GO:0046483	heterocycle metabolic process	2.50E-04
Biological process	GO:1901360	organic cyclic compound metabolic process	3.11E-04
Biological process	GO:0090304	nucleic acid metabolic process	4.20E-04
Biological process	GO:0044237	cellular metabolic process	0.001128
Biological process	GO:0009987	cellular process	0.002819
Biological process	GO:0006807	nitrogen compound metabolic process	0.031869
Molecular function	GO:0015453	oxidoreduction-driven active transmembrane transporter activity	1.35E-05
Molecular function	GO:0009055	electron transfer activity	1.76E-05
Molecular function	GO:0016779	nucleotidyltransferase activity	5.40E-05
Molecular function	GO:0140097	catalytic activity, acting on DNA	4.41E-04
Molecular function	GO:0015078	proton transmembrane transporter activity	7.51E-04
Molecular function	GO:0015399	primary active transmembrane transporter activity	0.001355
Molecular function	GO:0140640	catalytic activity, acting on a nucleic acid	0.002352
Molecular function	GO:0015318	inorganic molecular entity transmembrane transporter activity	0.003728
Molecular function	GO:0005215	transporter activity	0.004151
Molecular function	GO:0022890	inorganic cation transmembrane transporter activity	0.004449
Molecular function	GO:0008324	monoatomic cation transmembrane transporter activity	0.006613
Molecular function	GO:0003676	nucleic acid binding	0.006972
Molecular function	GO:0022857	transmembrane transporter activity	0.007882
Molecular function	GO:0015075	monoatomic ion transmembrane transporter activity	0.01248
Molecular function	GO:0046983	protein dimerization activity	0.013013
Molecular function	GO:0046914	transition metal ion binding	0.015856
Molecular function	GO:0022804	active transmembrane transporter activity	0.019202
Molecular function	GO:0016491	oxidoreductase activity	0.020779
Molecular function	GO:0008270	zinc ion binding	0.022801
Molecular function	GO:0097159	organic cyclic compound binding	0.032302

## Data Records

The *A. tanguticus* genome project has been deposited in the NCBI database under BioProject accession PRJNA1018692. The genome assembly and gene annotation have been deposited at GenBank under the WGS accession JAVYJV000000000^48^. The genomic Illumina sequencing data were deposited in the SRA at NCBI SRR26127850^49^. The nanopore sequencing data were deposited in the SRA at NCBI SRR26213735^50^. The Hi-C sequencing data were deposited in the SRA at NCBI SRR26152880^51^. The transcriptomic sequencing data were deposited in the SRA at NCBI SRR26156612–SRR26156618^52–58^.

## Technical Validation

### Evaluation of the genome assembly

The quality of the genome assembly of *A. tanguticus* was evaluated based on the contiguity, completeness, and correctness. For contiguity, Hi-C interaction analysis showed apparent interactions among the 24 pseudochromosomes, which was consistent with the reported chromosomes numbers of *A. tanguticus*^59^. Moreover, 97.47% of the draft genome sequences were oriented and ordered in the 24 pseudochromosomes, with a N50 of 51.28 Mb, suggesting a high contiguity of this genome assembly. For completeness, 97.83% complete BUSCO (benchmarking universal single-copy orthologs) genes in the genome assembly of *A. tanguticus* were retrieved by BUSCO (version: 5.2.2) analysis with embryophyta_odb10 database^60^. Additionally, the fragmented and missing BUSCO genes accounted for only 0.25% and 1.92%, respectively. For correctness, all Illumina short reads were mapped to the genome assembly by BWA^61^, with a high map rate of 99.96% in the genome assembly. Overall, the quality of the genome assembly was assessed as high contiguity, completeness, and correctness.

### Evaluation of the gene repertoire

The final gene repertoire of *A. tanguticus* comprised 44,252 protein-coding genes, while 38,388 or 38,128 protein-coding genes were predicted in the genome of *A. acutangulus*^10,62^. Given the phylogenetic proximity of *A. tanguticus* and *A. acutangulus* (Fig. 4), we compared the gene repertoires of these two species, focusing on both syntenic genes and non-syntenic genes. For syntenic genes, 34,447 genes in *A. tanguticus* genome corresponded to 33,162 genes in *A. acutangulus* genome (Table 7). For non-syntenic genes, 9,805 and 4,966 genes were predicated in *A. tanguticus* and *A. acutangulus* genome, respectively. The difference of gene repertoires of these two species mainly stemmed from the non-syntenic genes, which could result from the potential species-specific genes’ variation or a more detailed annotation of protein-coding gene in the *A. tanguticus* genome.

**Table 7:** The differences in gene repertoires of *A. tanguticus* and *A. acutangulus*.

Synteny gene number					Non-synteny gene number				
*A. tanguticus*		*A. acutangulus*		Difference	*A. tanguticus*		*A. acutangulus*		Difference
Chr1	2,161	Chr1	2,095	66	Chr1	472	Chr1	285	187
Chr2	1,422	Chr2	1,331	91	Chr2	379	Chr2	245	134
Chr6	1,956	Chr3	1,891	65	Chr6	397	Chr3	247	150
Chr4	1,624	Chr4	1,565	59	Chr4	503	Chr4	215	288
Chr5	1,506	Chr5	1,435	71	Chr5	458	Chr5	234	224
Chr3	2,052	Chr6	1,967	85	Chr3	528	Chr6	266	262
Chr7	1,364	Chr7	1,318	46	Chr7	371	Chr7	167	204
Chr8	1,369	Chr8	1,297	72	Chr8	374	Chr8	186	188
Chr9	1,746	Chr9	1,720	26	Chr9	394	Chr9	217	177
Chr11	1,369	Chr10	1,284	85	Chr11	337	Chr10	193	144
Chr10	1,225	Chr11	1,202	23	Chr10	346	Chr11	197	149
Chr12	1,313	Chr12	1,267	46	Chr12	362	Chr12	171	191
Chr18	1,791	Chr13	1,732	59	Chr18	359	Chr13	237	122
Chr13	1,384	Chr14	1,294	90	Chr13	302	Chr14	163	139
Chr15	1,406	Chr15	1,398	8	Chr15	381	Chr15	189	192
Chr14	1,182	Chr16	1,111	71	Chr14	355	Chr16	245	110
Chr17	1,151	Chr17	1,103	48	Chr17	283	Chr17	172	111
Chr16	1,681	Chr18	1,619	62	Chr16	327	Chr18	240	87
Chr19	1,312	Chr19	1,285	27	Chr19	375	Chr19	199	176
Chr21	1,184	Chr20	1,127	57	Chr21	307	Chr20	165	142
Chr22	926	Chr21	885	41	Chr22	268	Chr21	167	101
Chr23	1,440	Chr22	1,387	53	Chr23	327	Chr22	196	131
Chr20	911	Chr23	887	24	Chr20	320	Chr23	179	141
Chr24	965	Chr24	948	17	Chr24	293	Chr24	136	157
scaffold	7	scaffold	14	−7	scaffold	987	scaffold	55	932
	34,447		33,162	1,285		9,805		4,966	4,839

## Data Availability

The software and code used are publicly accessible. No custom programming or coding was used.
